# Digital teaching competencies and disability. Validation of a questionnaire design using the K coefficient to select experts

**DOI:** 10.1016/j.heliyon.2023.e16467

**Published:** 2023-05-22

**Authors:** José Fernández Cerero, José María Fernández Batanero, Julio Cabero Almenara

**Affiliations:** Department of Teaching and Educational Organization, University of Seville, Spain

**Keywords:** ICT skills, Disability, Questionnaire, Teacher training, Higher education, Expert competence

## Abstract

Higher education is one of the types of education most influenced by digital technologies. This situation, in educational contexts of quality and equity, presents different advantages but, at the same time, also many challenges. One of them is the use of ICT to support students with disabilities. In this line, the main objective of this study is to evaluate an instrument to measure the level of training and knowledge of university teachers in Spain concerning the application of ICT as support for students with disabilities. For the validation of content, the technique of “expert judgement” was used, applying a process of expert selection called “Expert Competence Coefficient” or “K Coefficient”. The instrument's reliability index was obtained through two statistics, Cronbach's alpha and McDonald's Omega. From the results obtained, it is confirmed that the questionnaire under study is an instrument with evidence of validity and reliability that allows us to diagnose, among university teaching staff, sub-dimensions of special relevance to find out the level of training and knowledge they have about ICT and students with disabilities.

## 1 Introduction

The technological revolution in which we are immersed entirely affects the field of education, opening up great opportunities for improving the quality, accessibility and equity of education [1]. Thus, digital competence is a requirement for the professional profile of teachers, especially if we consider that the application of technologies requires constant training of teachers. Moreover, digital competence is critical in designing, implementing and evaluating actions aimed at understanding and improving the education of a generation of students who are digital natives [2]. Likewise, improving digital competence is seen as a guarantee of successful teaching quality.

Despite the existence of quality practices linked to the use of ICT and a growing interest in the educational use of these technological tools, there still needs to be more concern about resolving the various difficulties in digital competence presented by teachers. Previous research findings argue that teachers must be sufficiently prepared to include technology effectively in their daily practice due to its instrumental use [3,4]. Even less so when it comes to including technology as a support for students with disabilities [5]; in this line, it is necessary to establish the difference between what is considered to have technical ICT skills and/or to be competent to put them into practice from a pedagogical perspective. Thus, ICT competencies include possessing the digital skills and attitudes to make integrated and functional use of knowledge [6]. How teachers can measure these ICT competencies is a complex task. Many researchers have focused on developing instruments that measure the use, approach and purpose of addressing classroom digital competence.

However, at present, studies on digital competence in teaching and disability are very scarce, and this means that one of the great difficulties we face is the lack of valid and reliable instruments that measure the level of knowledge and skills of university teaching staff for the application of ICT in this type of student body.

Therefore, considering the above comments, this article aims to present the validity process of a proposal to design an instrument to collect information on the technological competencies of university teachers in Andalusia (Spain) to support students with disabilities. The content validity process uses the so-called expert competence or “k" coefficient.

It is important to mention that this work is part of a series of research results related to developing a training plan. In this sense, our study is important because a good diagnosis of the level of technical competencies of teachers will contribute to the improvement in the design and elaboration of inclusive training plans. This will improve education through good teaching practices with all students, as recognised in Article 24 of the Convention on the Rights of Persons with Disabilities, which the Kingdom of Spain signed and ratified on May 3, 2008. At the same time, this perspective favours the achievement of the Sustainable Development Goals established by the UN for the year 2030, precisely the fourth goal of “Ensure inclusive and equitable quality education and promote lifelong learning opportunities”. Therefore, the scarcity of work in this area of knowledge motivated the present study, which opens up an emerging line of research in the context of a university marked by educational inclusion. The main objective of this study is to evaluate an instrument to measure the level of training and knowledge of university teaching staff in Spain regarding the application of ICT to support students with disabilities.

## 2 Theoretical and methodological background

People with disabilities are one of the groups in higher education that have faced the most significant barriers to learning and participation. Studies conclude that many students with disabilities fail to complete their university studies [7]. In this regard, Moriña [8], in his literature review, reported that inaccessibility to ICT was identified by students with disabilities pursuing higher education as one of the barriers they encounter. Similarly, research has identified teachers' pedagogical and technological competencies as a key to improving the quality of teaching and learning in higher education [9]. However, good teacher training requires a study to be carried out to ascertain the reality from which it starts. This necessarily involves the creation of valid and reliable instruments that allow for diagnosis. Tools referring to the knowledge and digital competence of teachers and students on ICT and disability have been developed, some of them by researchers who are signatories to this article [[10], [11], [12]]. Instruments that aim to collect information from teachers on their knowledge and level of training in “assistive technologies” designed to improve autonomy, functional capacity and accessibility to knowledge. Thus, when we talk about “assistive technologies” in higher education for people with disabilities, we are referring to accessibility; assistive technologies for people with physical disabilities, visual disabilities, hearing disabilities; cognitive disabilities and learning disabilities [13].

Although research in this direction and in the international context is minimal [9,14], research has been conducted that also incorporates knowledge of accessible learning and assistive technologies for people with sensory disabilities as a means in their training programmes to increase inclusive digital competence [[15], [16], [17]].

In the Spanish context, the studies carried out have gone in the same direction, thus the work of Cabero-Almenara, Fernández-Batanero, & Cordoba Pérez [10] aimed at the construction and validation of a diagnostic instrument developed to find out the level of training and knowledge, of future teachers in Spain, regarding the application of ICT for people with disabilities. The questionnaire comprised 79 items divided into 6 dimensions (general aspects, visual impairment, hearing impairment, cognitive impairment, motor impairment and accessibility). These dimensions focus on the different sensory disabilities. An adaptation of this instrument was carried out by Medina et al. [18] to find out the inclusive digital knowledge of teachers at all educational stages. The dimensions used were the same as in the original instrument.

In a later study focusing on primary and secondary school teachers, the level of digital competencies as a support for students with disabilities was also studied. This research reports that the questionnaire comprised 53 items divided into 6 dimensions (general aspects, visual impairment, hearing impairment, cognitive impairment, motor impairment and accessibility) [11]. Subsequently, in another study aimed at finding out the level of digital competence of early childhood, primary and secondary education teachers, another questionnaire was also developed and validated, consisting of 31 items that sought to collect information on general aspects of the application of ICT for people with disabilities and specific elements for the application of ICT for people with motor, cognitive, visual, hearing impairments and general accessibility awareness [12]. This study emphasised the identification of factors using logistic regression methods.

In a recent literature review research [19] that aimed to study existing work on increasing teachers' competence in providing accessible and inclusive digital learning environments and materials to higher education students, it is reported that there is a lack of commonly accepted instruments to assess training outcomes. Future research should focus on establishing a standard tool and objective measures to evaluate training outcomes.

Thus, considering the above, we estimate a gap in creating valid and reliable instruments for teacher diagnostics related to digital competences and disability. Hence, the contribution of our work is relevant for the scientific community.

## 3 The use of the expert method: the coefficient of expert competence (k)

In the scientific literature, expert judgement is recognised as the method that provides assessments to determine the relevance and feasibility of proposals [20]. From this perspective, “Expert consultation constitutes a heuristic method of high scientific rigour that allows the search for consensus based on qualitative approaches derived from the experience and knowledge of a group of people” [21]. In addition, there are journals with a high level of visibility indexed in essential databases, where works have been published that implement the Coefficient k in fields as diverse as medicine [21], education [22,23], agronomy [24], computer science [25], among others. This denotes qualitative evidence about the importance given to this coefficient and its applications, mainly in the field of social sciences in the last five years [26].

The use of the K-value has the following advantages: the theoretical quality of the answers obtained, the level of depth of the solutions obtained, and the ability to collect detailed information [27]; at the same time, it is considered a beneficial technique for establishing the knowledge of the art in difficult, complex, novel or little studied contents and topics [28]. Other authors, such as Hurtado de Mendoza [29], consider the following advantages: it is based on the assumption that several experts can arrive at a better forecast than a single person; there is no secrecy and communication is encouraged because sometimes forecasts and validations are influenced by social factors and may not reflect a consensus; and finally, as a visionary forecast it is a prophecy that uses unique ideas and judgement, linked to each other.

On the other hand, there are several problems associated with the use of the expert method, which is related to the very concept of expert, as it is a rather polysemic term and is usually associated with how practical, skilled or experienced we can consider a person to be [26]. As Mengual [30] indicates, we can understand an expert as: “both the individual and the group of people who can provide reliable assessments of a problem in question, and at the same time, make recommendations based on a maximum level of competence”. Another aspect related to the restrictions of the K-value lies in the apparent simplification that balances knowledge and sources of argumentation in the form of an arithmetic average. This aspect, for some authors, requires research at the theoretical and practical levels [31]. For his part, Hurtado de Mendoza [29] considers that the disadvantages of this methodology include, on the one hand, its lack of security, too much sensitivity of the results to the ambiguity of the questions, and on the other hand, he considers that qualitative methods rely mainly on the judgement of experts and tend to be less precise than quantitative methods. Despite these limitations and taking into account their positive qualities, their current use in organisations and research suggests that their potential often exceeds their limits.

In any case, the studies defend the search for an integrated approach to the problem and the need for effective selection instruments based on indicators that capture the essential features of expert competence [26]. In this sense, studies agree that the results obtained depend directly on the quality of the experts selected for the evaluation process, and for this, different procedures range from considering the profile of the chosen expert to other, more complex ones, such as the coefficient of expert competence [22]. Ultimately, the quality of the results obtained through expert judgement will be influenced by people's choices, experience, and knowledge of the subject. Therefore, to ensure that the people selected are genuine experts in a given field of research, it is recommended to calculate the Expert Competence Coefficient or K-Coefficient [32].

Considering the above comments, our starting hypothesis is: The K coefficient is an effective and valid instrument for assessing university lecturers' level of education and training about ICT applied to students with disabilities.

## 4 Methodology

This study aimed to design and validate an instrument to collect information on the level of technological skills of university teachers in Andalusia (Spain) to support students with disabilities.

The methodology used initially was content validation by experts. The expert competence coefficient “K" [26] was used for selection. The instrument's reliability was obtained through two statistics, Cronbach's alpha and McDonald's Omega [33,34].

### 4.1 Procedure

For the construction of the instrument, a series of phases were followed: a) literature review, b) development of the first version of the instrument and formulation of the items, c) application of the instrument to the “expert judgement” technique for its evaluation, d) development of the final version of the instrument, and e) pilot testing and obtaining the reliability index. In the literature review, we opted for an instrument previously developed in another research project [11] but set in the field of primary education. We first searched the literature to adapt the questionnaire to the university context. The second step was to adjust the items related to the different types of disability under study (visual, motor, hearing and intellectual) to the context of higher education and the items related to “Accessibility”. An additional dimension (Services) was also added. We considered it necessary to add the dimension “Services” because the “Units of Attention to People with Disabilities” of the Spanish universities are included in the Spanish university legislation [35]. These units constitute excellent support for the full inclusion of these students in higher education. The next step for its adaptation was the revision of the questionnaire by a group of 9 university teachers from the Departments of Didactics and Educational Organisation of the Universities of Seville and Granada, intentionally selected for convenience, according to previously established criteria that we considered significant, i.e., being a teacher specialised in Educational Technology and/or Special Education. After review and appropriate modifications, the questionnaire consisted of two blocks: The first aimed to collect information on different biographical aspects of teachers: gender, age, years of teaching experience, professional category, higher education institution where they teach, etc. The aim was to obtain sufficient information to allow us to obtain enough information to improve the quality of teaching. The objective is to obtain information necessary to justify the selection of the expert, inferring the suitability and relevance for the requested activity.

The second block comprises 61 items, which aim to collect information on the teacher's assessment of their technical-teaching mastery of different technologies and the evaluation of general aspects related to the application of ICT for students with disabilities. This part of the instrument consists of a Likert-type scale with 6 scores where value 1 refers to “you feel completely ineffective”, and value 6 refers to “I master it completely".

The final questionnaire comprised 62 items divided into 7 dimensions (General, Visual, Auditory, Motor, Cognitive, Accessibility and Services). No open questions were included. Table 1 presents the dimensions and associated items.

**Table 1:** Dimensions and associated items.

Dimensions	Items
General	1,2,3,4,5,6,7,8,9,10,11,12
Visual	13,14,15,16,17,18,19,20,21,22,23,24,25
Auditory	26,27,28, 29, 30, 31, 32
Motor	33,34,35,36,37,38,39,40
Cognitive	41,42,43,44,45,46,47,48,49
Accessibility	50,51,52,53,54,55,56
Services	57,58,59,60,61

The instrument was administered online and can be viewed at the following web address: https://cutt.ly/uhtYHdE.

**Fig. 1:**
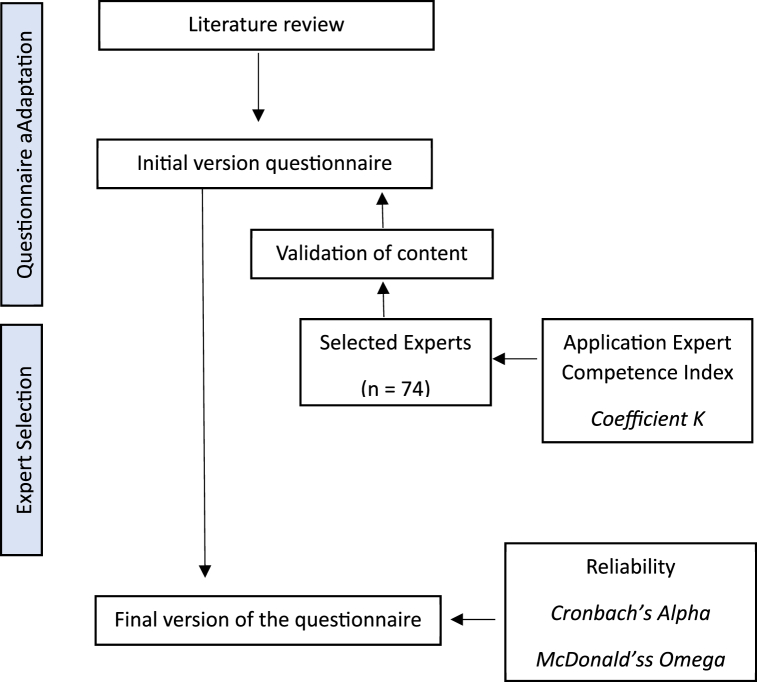
Questionnaire validation process.

Fig. 1 shows the process followed in the validation of the questionnaire.

Its significance for the objectives we were pursuing was assessed using expert judgement. A double procedure was applied for selecting experts: program and expert competence coefficient. Authors such as Cabero, Romero & Palacios [32] state that the importance of the selection of experts lies in the fact that, if valuable results are to be achieved for the objectives pursued, particular emphasis must be placed on their choice, and for this purpose, the combination of the biogram and the expert competence index is presented as an appropriate option.

In the present study, two expert selection mechanisms were established. Firstly, we identified them by taking into account that they met two or more of the following criteria.

• Teaching in Universities on the subjects of “Educational Technology”, “New Technologies applied to Education”, or “Information and Communication Technologies applied to Education”.
• Teaching in Universities on the subjects of “Inclusive Education”, “Learning Disability”, or “Special Education”.
• Have experience in the field of teacher training in ICT.
• Have experience in the field of teacher training in inclusive education.
• Have written an article on ICT and special education, diversity, or inclusive education.
• To have previously collaborated with us on other research projects, which would allow us to know their seriousness and professionalism.

One of the problems associated with the expert method is in relation to the number of experts needed for its application. In this sense, it must be said that the proposals of different researchers range from 15 to 20 [36], 15–25 [37], or 15–25 [38]. However, other researchers need to mention a consensus regarding the number of experts used [39]. In this regard, several authors recommend a number more significant than ten experts [40,41]. This minimum number provides an acceptable estimate of content validity, facilitating the detection and exclusion of outliers from the evaluator [42].

In our case, and given that we could count on enough experts, we decided to work with as many as possible. The database of experts was constructed from different sources: existing contacts of members of the research group, knowledge of the biographies of certain researchers, and their membership of the “Edutec Association” and the “Special Education Network”. Therefore, 135 e-mails were sent requesting their participation (Annex I), to which 116 university teachers replied affirmatively.

The second step was to apply the Expert Competence Coefficient (K) to these 116 teachers for their final selection [11,43,44]. For this purpose, the questions presented in Annex I was included in the questionnaire.

All subjects gave their informed consent for inclusion before they participated in the study. The study was conducted in accordance with the Declaration of Helsinki, and the fundamental principles of research integrity were respected in accordance with the Research Ethics Committee of the University of Seville.

It should be noted that the K Coefficient is obtained by applying the formula: K = ½ (Kc + Ka). In Kc is the “knowledge coefficient” obtained from the assessment offered directly by the expert in question An of Annex I and Ka. This argumentation coefficient is obtained through the options specified by the expert in the table in question B of Annex I. The “Argumentation Coefficient” (Ka) assesses the degree of significance that we can give to different aspects carried out by the expert (theoretical analyses carried out by the expert, studies carried out on the subject, study of works on the subject by foreign authors, etc.) for us to consider them as an expert. In colloquial terms, it would be like asking, “why do you rate yourself this way? Give me some evidence; do you say it because you have conducted many theoretical studies on the subject; why have you written a lot, etc.?

The values used to determine the expert's position are [26].

- 0,8 < K < 1,0 high competition coefficient
- 0,5 < K < 0,8 average competition coefficient
- K < 0,5 low competition coefficient

Therefore, only those judges or experts who obtained a score equal to or higher than 0.8 will be used in the present study, which led us to keep 74 (63.79%). In this sense and considering Dunning Kruger [45], a positive correlation between perceived and actual effect skills is evident.

The way we carried out our expert judgement was by individual aggregation of the experts, which consists of obtaining the information individually from each one, without there having to be contacted between them [46].

## 5 Results

Therefore, only those judges or experts who obtained a score equal to or higher than 0.8 will be used in the present study, which led us to keep 74 (63.79%).

**Fig. 2:**
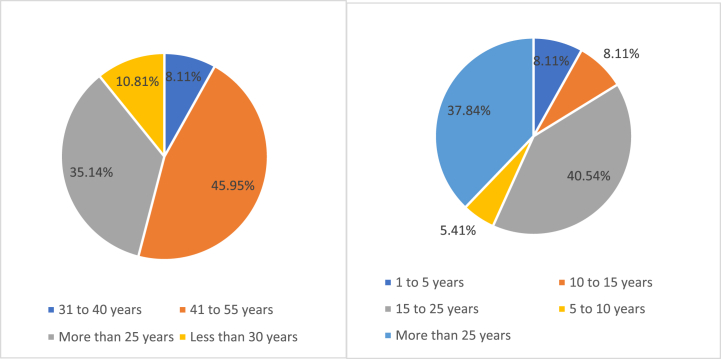
Age and experience of the experts.

The way we carried out our expert judgement was by individual aggregation of the experts, which consists of obtaining the information individually from each one, without there having to be contacted between them [46]. Fig. 2 shows the age and experience of the selected experts.

About their employment category, the vast majority (f = 46, 62.16%) were full university lecturers, followed by 10 (13.51%) who were contracted lecturers.

When asked whether, during their years of teaching experience, they had received information on issues related to the use of Information and Communication Technologies (ICT) applied to people with disabilities, 32 (43.24%) said that they had not, and 42 (56.76%) said that they had. Very similar data were obtained when asked whether, during your years as a teacher, you have received information on universal design and accessibility for the educational application of ICT, where 34 (45.95%) said no, and 40 (54.05%) said yes.

When the teachers were asked to rate themselves from 1 (not at all) to 10 (very much) about their mastery of different technologies, the values obtained were as follows (Table 2).

**Table 2:** Teachers' self-assessment of their mastery of different technologies.

	Mean	S.D.
How do you rate your training in the technical handling of ICTs?	8,14	1,46
How do you rate your training in the educational use of ICT?	8,08	1,30
How do you rate your training in the technical operation of the Internet?	8,14	1,29
How do you rate your training in the educational use of the Internet?	8,16	1,12
I believe that ICTs are a support resource for people with disabilities.	9,43	,80

Finally, the judges or experts were asked to assess the significance of the different items to be incorporated into the elaborated instrument and their appropriateness for the dimension in which they were included. According to a scale of six intervals ranging from MN=Very negative/very unwelcome (1) to MP=Very positive/very much agree (6). Table 3 shows an example of the mean scores and standard deviations obtained for some items. The remaining mean scores and standard deviations can be found in Annex II.

**Table 3:** Means and standard deviations were obtained from the different items of the instrument.

	Mean	S.D.
I have a general knowledge of the possibilities offered by ICT for people with disabilities.	5,19	0,70
I know the difficulties caused by different types of disabilities in using ICT.	5,16	0,76
Can select specific ICT according to different people's physical, sensory and cognitive characteristics.	5,03	0,80
I consider myself competent to locate educational materials for people with special educational needs online.	5,08	1,01
I am able to use sign language.	3,00	1,92
I am able to express messages according to sign language.	3,05	1,94
I am able to adapt computer equipment to the educational needs of any person with a disability.	3,97	1,59

Overall, the mean value obtained was 4.70, and the mean value of the standard deviation was 1.31. The results obtained indicate two main aspects: a) that except for two items, “I am able to use sign language” and “I am able to express messages according to sign language”, which obtained a score representing a rating of “Fair negative/moderately disagree”, the rest were rated as “Positive/Agree-relevant”, and b) and that some elevation has been found in the standard deviations obtained, suggesting some dispersion of data.

With the data obtained, we also calculated Gwett's AC Coefficient. Gwett's AC coefficient indicates the degree of agreement that includes a penalty for agreement by chance but is not affected by what has been called the Kappa paradox [47]. This paradox, which affects not only Cohen's Kappa but also other coefficients (Fleiss' Kappa, Krippendorf's Alpha), occurs when the distribution of expert judgements is highly skewed towards one of the categories. In such cases, despite a high percentage of agreement, Cohen's kappa and the other mentioned coefficients tend to be very low, sometimes leading to negative values [48]. In our case, we obtained an agreement percentage of 93.79%, i.e. 0.90, according to Gwett's CA coefficient. Therefore, the items that make up our modified questionnaire after this assessment have validity criteria for sufficiency, clarity, coherence and relevance.

The assessment of the items by the experts ensured an adequate level of content validity of the instrument.

Additionally, we wanted to test the instrument's reliability, which was obtained through two statistics, Cronbach's alpha and McDonald's Omega [33,34], the values obtained are presented in Table 4.

**Table 4:** Reliability index.

Dimension	Alpha	Omega
General	0.965	0.970
Visual	0.975	0.978
Auditory	0.968	0.971
Motor	0.974	0.979
Cognitive	0.979	0.981
Accessibility	0.958	0.959
Services offered University	0.954	0.960
Global	0.991	0.998

Note: It should be noted that the total item correlation was carried out to see if eliminating any item increased the instrument's reliability, which did not occur.

According to O'Dwyer & Bernauer [49], the values obtained indicate high levels of reliability, both for the instrument as a whole and for its different dimensions.

## 6 Limitations

The strength of our study has been to present a compelling and valid instrument to assess the level of training and competencies of university lecturers about ICT applied to students with disabilities. However, despite this, we would like to point out some limitations of this study that may condition the use and application of the scale we present. In this sense, a single index can provide a limited measure of a very complex set. In our case, we propose to estimate the accuracy of the expert measure by contrasting its results with those obtained by other methods. Developing new research that goes deeper into theory and experimental practice is necessary. The variables to be compared could be the underlying models, the degree of the capture of the concept of expertise, levels of reliability and validity, and cut-off points, among others.

## 7 Discussion and conclusions

With this work, we wanted to provide a valuable and useful tool to measure the level of digital competence of university teaching staff to support students with disabilities. Our purpose has been to provide a measure that allows us to generate useful and applied knowledge about ICT and disability, focusing on key aspects and assistive technology. Although much has been written and reflected on ICT and disability [7,8,12], few studies have provided measures in this area of knowledge. With this study, we wanted to give a valuable and valid tool for diagnosing digital competence in teaching applied to disability. This effectiveness is demonstrated because it has been used previously to construct diagnostic instruments in digital competencies applied to different problems [5,10,50].

Without detriment to other dimensions that may be equally relevant for the study of digital competence about student disability, in this paper, we document the relevance of strategies and resources as critical dimensions in the learning of ICT in students with sensory disabilities. These dimensions repeatedly appear in the recent scientific literature [11,12].

The questionnaire on digital competencies and disability that we have designed is sensitive to measuring technical training not only as a support for the different types of sensory disabilities but also the resources on which learning is based in these. In this way, the questionnaire allows us to investigate aspects related to accessibility, showing the resources on which learning is supported by assistive technology.

In short, two main conclusions have been obtained. The first refers to the effectiveness shown by the expert selection procedure through the expert competence coefficient, which allows the selection process to be fine-tuned, as stated in the literature [26]. However, it should be combined with a prior expert identification procedure, such as the one used in this paper. And secondly, the assessments made by the experts and the reliability indices of the instrument allow us to indicate the validity and reliability of the instrument.

About the added value of our study, it must be said that it provides knowledge of teachers' competence in providing accessible and inclusive digital learning environments and materials to higher education teachers. The results obtained from this questionnaire can be used to adapt the most suitable training strategies to promote the learning of students with disabilities in higher education.

This research opens the door not only to future studies on teacher diagnosis about ICT learning and students with disabilities. It also opens the possibility of reflection for establishing a standard instrument and objective measures to evaluate the training results.

## Funding

This work was partially suported by the project I + D + i, PID2019-108230 R B-I00, funded by MCIN/AEI/10.13,039/501,100,011,033.

## Author contribution statement

José Fernández Cerero; José María Fernández Batanero; Julio Cabero Almenara: Conceived and designed the experiments; Performed the experiments; Analyzed and interpreted the data; Contributed reagents, materials, analysis tools or data; Wrote the paper.

## Data availability statement

Data included in article/supp. Material/referenced in article.

## Additional information

No additional information is available for this paper.

## Declaration of competing interest

The authors declare that they have no known competing financial interests or personal relationships that could have appeared to influence the work reported in this paper

## Figures and Tables

**Table:** 

0									10
									

(Note: The Kc score (Knowledge Coefficient - value from 0 to 10) is obtained).

**Table:** 

	Low	Medium	High
Theoretical analysis carried out by you			
Your experience gained from your practical activity			
Working study on the subject by Spanish authors.			
Study of work on the subject by foreign authors.			
Your knowledge of the state of the problem abroad			
Your intuition about the subject matter.			

**Table:** 

	Mean	S.D.
I have a general knowledge of the possibilities offered by ICT for people with disabilities.	5,19	0,70
I know the difficulties caused by different types of disabilities in using ICT.	5,16	0,76
Can select specific ICT according to different people's physical, sensory and cognitive characteristics.	5,03	0,80
I am aware of different resources and documents specifically dedicated to analysing the possibilities of ICT for people with different types of disabilities.	4,97	0,80
I am aware of educational experiences in applying ICTs for people with different types of disabilities.	5,22	0,71
I know of mobile applications which can be used with people with special educational needs.	4,70	0,94
I know Augmented/Virtual Reality applications that can be used with people with special educational needs.	4,41	1,34
I know what 3D printing technology can do to help people with special educational needs.	4,51	1,33
I am aware of the main limitations that may condition the use of ICT by learners with disabilities.	4,7	1,27
I consider myself competent to locate educational materials for people with special educational needs online.	5,08	1,01
In general, I feel prepared to help learners with certain disabilities in the use of technical aids and the use of ICT.	4,86	0,86
I can design activities with generalised educational software for students with special educational needs.	4,46	1,28
I am able to explain the possibilities offered by a Braille typewriter.	4,54	1,35
I know the possibilities offered by Kurzweil reading machines for visually impaired students.	4,38	1,50
I am aware of the possibilities offered by telescopes for visually impaired pupils.	4,78	1,03
I recognise different computer programmes specifically designed for visually impaired people.	4,86	1,08
I am familiar with different screen magnification programmes to facilitate access to the computer for visually impaired students.	4,73	1,10
I know different screen reader softwares, such as JAWS, Tiflowin, etc ….	4,65	1,14
I can produce didactic materials in a word processor, eliminating the aspects that make it difficult for visually impaired people to use it.	4,72	1,02
I am able to list different tiflotechnological materials that enable visually impaired people to access numeracy.	4,59	1,21
I know of specific browsers for visually impaired people.	4,3	1,54
I am aware of different websites where educational resources for people with visual impairment can be found.	4,73	1,22
I am aware of the possibilities that ICT offers the visually impaired student.	4,81	1,22
I am aware of the possibilities that 3D printers offer to visually impaired students.	4,65	1,30
I am able to use sign language.	3,00	1,92
I am able to express messages according to sign language.	3,05	1,94
I am able to identify different computer resources for voice and speech enhancement.	4,3	1,60
I am familiar with different educational software programmes that stimulate language and the acquisition and development of oral and written language skills.	4,32	1,38
I am able to identify different websites where educational resources for people with hearing impairment can be located.	4,81	1,15
I am able to apply teaching strategies supported by ICT to facilitate the inclusion of students with hearing impairment.	4,73	1,22
I am able to make ICT-supported curricular adaptations for students with hearing impairment.	4,73	1,19
Generally speaking, I am aware of the possibilities that ICT offers to hearing impaired learners.	4,65	1,25
I am familiar with different speech re-education programmes.	4,24	1,40
I know different types of keyboards for people with different types of mobility limitations.	4,51	1,54
I know the uses of switches, toggles and pointers.	4,65	1,50
I know of software that controls the computer by voice.	4,76	1,31
I know the basics of alternative augmentative software systems to facilitate communication for people with motor disabilities.	4,57	1,28
I locate websites containing educational resources for people with motor disabilities.	4,92	1,21
I am able to apply ICT-supported teaching strategies to facilitate the inclusion of learners with motor impairments.	4,89	1,20
I am aware of the possibilities offered by ICT for students with motor disabilities.	4,78	1,25
I am aware of the possibilities that 3D printers are offering for the manufacture of prostheses.	4,68	1,47
I can cite some educational programmes used for the rehabilitation of cognitive skills.	4,78	1,25
I am able to cite different websites where educational resources for people with cognitive disabilities can be located.	4,71	1,33
I can use specific software to produce materials for a concept keyboard.	4,16	1,52
I am able to apply ICT-supported teaching strategies to facilitate the inclusion of learners with cognitive disabilities.	4,72	1,39
I am able to make ICT-supported curricular adaptations for subjects with cognitive disabilities.	4,78	1,29
I am able to describe the main limitations that multimedia materials may contain for use with people with cognitive disabilities.	4,62	1,34
I can locate websites that contain educational resources for people with cognitive disabilities.	4,81	1,22
I am aware of the possibilities offered by ICT for students with cognitive disabilities.	4,78	1,29
I know the potential offered by Apps or mobile applications dedicated to cognitive training or rehabilitation of people.	4,81	1,29
I am aware of the possibilities offered by operating systems and browsers to modify certain levels of programme operation (speed, font size, pointer type, etc.) and make the programme more accessible to people with different types of disabilities.	4,62	1,44
I know what accessibility testing is for websites.	4,86	1,23
I am familiar with the general WAI/W3C guidelines for making websites accessible.	4,57	1,37
I am able to create web pages with high accessibility parameters.	4,08	1,55
I am able to adapt computer equipment to the educational needs of any person with a disability.	3,97	1,59
I know different institutions that are involved in the study and research of website accessibility.	4,68	1,18
I am able to cite different accessibility tests.	4,35	1,48
I am aware of the services that my university offers for people with disabilities.	4,73	1,50
If I have a student with a hearing impairment I know where in my university I can direct them.	4,76	1,67
I am aware of the services offered in my faculty for people with special needs.	4,84	1,57
I know the services my university offers to people with motor impairments.	4,73	1,56
I know the services my University offers to people with visual impairment.	4,78	1,60

## Annex I

### Dear teacher and researcher

I am José Fernández Cerero and I am carrying out a study that is part of a more comprehensive R + D + i research project (PID2019-108230 R B-I00/10.13039/501,100,011,033). The study aims to increase the training of higher education teachers in ICT and disability.

To do so, we need to collect information on the level of training and technological knowledge that the Research Teaching Staff teaching in the Faculties of Education of the different Universities of the Autonomous Community of Andalusia have regarding the application of ICT for people with different types of disabilities.

We ask you to answer honestly to select the experts for the questionnaire.

A) Tick in the appropriate box the degree of knowledge you have about the following topics: teacher training in ICT, ICT and inclusive education, disability, accessibility, ICT and disability, …. Please rate yourself on a scale of 0–10 (with 0 being no knowledge at all and 10 being full knowledge of state of the art).
B)Self-assess the degree of influence that each of the following sources has had on your knowledge and views on teacher training in ICT, ICT and inclusive education, disability, accessibility, ICT and disability, ….
